# Therapist competence, therapy quality, and therapist training

**DOI:** 10.1016/j.brat.2011.03.005

**Published:** 2011-06

**Authors:** Christopher G. Fairburn, Zafra Cooper

**Affiliations:** Oxford University Department of Psychiatry, UK

**Keywords:** Psychotherapy, Psychological treatment, Training, Competence, Internet, Dissemination

## Abstract

Large numbers of therapists worldwide wish to receive training in how to deliver psychological treatments. Current methods of training are poorly suited to this task as they are costly and require scarce expertise. New forms of training therefore need to be developed that are more cost-effective and scalable. Internet-based methods might fulfil these requirements whilst having the added advantage of being able to provide trainees with extensive exposure to the treatment as practised. New strategies and procedures for evaluating training outcome are also required. These need to be capable of assessing the therapist’s knowledge of the treatment and its use, as well as the therapist’s ability to apply this knowledge in clinical practice. Standardised role play-based techniques might be of value in this regard.

## Introduction

It is remarkable that in this era of enthusiasm for evidence-based psychological treatments so little research attention has been paid to therapists’ ability to deliver these treatments. Training methods have changed little over the years and the measurement of the outcome of training has been largely overlooked. In this two-part article these inter-related topics will be considered, starting with therapy quality and therapist competence.

## Part One – Therapy Quality and Therapist Competence Why therapist competence is important

There are at least three reasons why therapists’ ability to deliver psychological treatments is important. The first relates to the responsibility of all clinicians to provide their patients with the best possible care or treatment. For those of us who are psychotherapists^1^, this means delivering appropriate psychological treatments in a competent manner. The second reason stems from the pressing need to disseminate evidence-based psychological treatments (Barlow, Levitt, & Bufka, 1999; McHugh & Barlow, 2010). To do so requires training large numbers of therapists to the point at which they are “competent” but what this means and how it should be assessed has received little attention (Sharpless & Barber, 2009). The third reason arises from concerns about validity of the research on psychological treatments. To draw firm conclusions from the studies of the efficacy and effectiveness of psychological treatments it is essential that the treatments were delivered competently, yet the true standard of implementation of psychological treatments is rarely assessed (Perepletchikova, Treat, & Kazdin, 2007).

## Therapy quality

Various terms and concepts have been used when referring to the standard of implementation of a psychological treatment, the best known being *treatment fidelity* or *treatment integrity*. These terms were introduced by treatment researchers to refer to the extent to which a treatment was implemented as intended. Two aspects of treatment fidelity (integrity) are often distinguished (e.g., Perepletchikova & Kazdin, 2005; Waltz, Addis, Koerner, & Jacobson, 1993). The first is treatment *adherence*. This is whether the right psychotherapeutic procedures were used. A parallel in surgery would be selecting the optimal operative procedures. The second notion is that of *competence*. This refers to how well the chosen procedures were implemented. The surgical parallel would be performing the operative procedures in a skilful manner. A third notion, that of *treatment differentiation*, refers to whether the distinctiveness of the psychological treatment was maintained by not including extraneous and possibly proscribed elements. This last concept (which can be subsumed under adherence) is central to the experimental validity of studies in which psychological treatments are being compared.

While these distinctions are meaningful and of value in the context of treatment research, they seem less pertinent to everyday clinical practice. This particularly applies to the distinction between adherence and competence which is of little consequence if the matter of concern is the overall standard of the treatment provided; for example, high adherence is of little interest in the presence of low competence (i.e., doing the right things badly), and vice versa (i.e., doing the wrong things well). What matters is doing the right things well. We therefore favour abandoning these distinctions when referring to routine clinical practice, and instead adopting a broader notion, one of “*therapy quality*”, defined as “the extent to which a psychological treatment was delivered well enough for it to achieve its expected effects”^2^. Thus the term therapy quality refers to the standard of delivery of a particular course (or session) of treatment.

## Therapist competence

Therapy quality needs to be distinguished from *therapist competence*, the latter notion referring to an attribute of a therapist, not a treatment. Therapist competence in this context may be defined as “the extent to which a therapist has the knowledge and skill required to deliver a treatment to the standard needed for it to achieve its expected effects.” Thus when assessing therapist competence one is assessing the therapist’s capacity to provide a treatment to an acceptable standard. This requires evaluating the therapist’s knowledge of the treatment and its use, and the therapist’s ability to implement it. This particular type of competence has been described as “*limited-domain intervention competence*” (Barber, Sharpless, Klostermann, & McCarthy, 2007) as it refers to the therapist’s ability to implement a specific form of treatment. Psychotherapists have to possess a range of other abilities; for example, more global psychotherapeutic ones (Barber et al., 2007; Sharpless & Barber, 2009), the ability to assess patients well, and the ability to select treatments appropriately. These “competencies” are outside the scope of the present article.

## Methods used to assess therapist competence and therapy quality

There has been very little research on the assessment of therapist competence and therapy quality. The main quantitative methods are discussed below.

### Direct measures of knowledge

The assessment of a therapist’s knowledge of a treatment and its use is central to the evaluation of therapist competence. The body of knowledge that needs to be assessed is extensive: it is more than merely knowing the treatment’s strategies and procedures. It embraces its indications and contraindications; whether it can (or should) be combined with other forms of treatment, including pharmacotherapy; typical responses to the treatment, and any adverse effects; common difficulties encountered, and how to address them; and when to abbreviate or extend the treatment, and when to abandon it. It also includes knowing what not to do, a topic that is sometimes neglected; for example, not doing things that might interfere with the therapeutic relationship (e.g., ignoring the patient’s concerns about the treatment) and not doing things that might undermine the treatment (e.g., by introducing elements from other treatments that might confuse or distract the patient).

Most of the studies of therapist training (reviewed by Herschell, Kolko, Baumann, and Davis (2010) and Rakovshik and McManus (2010)) have included a knowledge test, but the measures employed have tended to be narrow in focus, concentrating largely on the treatment’s strategies and procedures. As far as we are aware, no standardised knowledge tests have been developed.

### Measures of skill at implementing a treatment

Also central to the assessment of therapist competence is the evaluation of the therapist’s ability to implement the treatment (i.e., their ability to apply their knowledge to clinical practice). Three main methods are used.

#### The evaluation of patient outcome

At first sight this is a compelling index as the goal of treatment is to benefit patients. In practice, however, it is problematic. Its main shortcoming is that it is an indirect measure as patient outcome is affected by variables other than the quality of the treatment provided, a key one being the characteristics of the patients in question. Patients vary in their responsiveness to treatment (e.g., as a result of differences in the severity or duration of their problems, the extent of any comorbidity, and the presence of complicating life circumstances) and unless this is taken into account when evaluating outcome data a false impression may be obtained. This is exemplified by the research on the use of mortality data to assess the competence of cardiac surgeons where it has emerged that most of the variability between surgeons is attributable to differences in the proportion of high risk patients treated (Bridgewater et al., 2003; Bridgewater & Keogh, 2008). Therefore crude outcome data can be misleading, and this is especially the case if data are only available on a handful of patients, as is often the situation with psychological treatments. A treatment service may be evaluated in terms of patient outcome, but not individual courses of treatment or, indeed, individual therapists except in those unusual instances where there is high patient throughput as in certain treatment trials and implementation initiatives.

#### The evaluation of treatment sessions

A more widely used method for assessing the skill of therapists is the evaluation of the quality of their treatment sessions (i.e., therapy quality is being used as an index of therapist competence). This therapy quality method requires that treatment sessions be evaluated using a standard procedure. In the field of cognitive behaviour therapy (CBT), for example, common practice is for treatment sessions to be rated using the Cognitive Therapy Scale (CTS; Young & Beck, 1980, 1988) or its revised version (CTS-R; Blackburn, James, Milne, & Reichelt, 2001). These measures require that treatment sessions (usually recordings of them) be evaluated by a rater with respect to the presence and quality of certain therapist-determined features (e.g., the eliciting of key cognitions, the use of guided discovery, the setting of homework). On this basis a score is generated and if it is above a specified threshold the session is judged to have been delivered sufficiently well (i.e., competently).

In principle, this is an attractive way of evaluating therapist competence as it directly assesses the therapist’s performance at implementing a treatment. In practice, however, it is problematic. For example, it has proved difficult to define, operationalise and demarcate the aspects of treatment of interest with the result that inter-rater reliability has been less than satisfactory (e.g., Jacobson & Gortner, 2000). Even more challenging has been the matter of validity. It has not been established that the various therapy rating scales measure what they purport to measure, nor has it been demonstrated that their threshold scores are appropriate ones for viewing sessions as having been delivered well enough.

There is another shortcoming. This concerns the features assessed. For example, the CTS and CTS-R focus largely on aspects of treatment that are common to most forms of CBT and, of necessity, ones that are expected to be present in most treatment sessions. They do not assess the disorder-specific strategies and procedures that are viewed as being central to the mode of action of most evidence-based forms of CBT. Thus they are more measures of the CBT “style” of conducting treatment than measures of any specific form of CBT.

An additional matter of importance is that there is rarely a formal protocol for the use of these instruments. Three matters are of concern. First, few sessions tend to be rated as doing so is time-consuming. As a result, generalisations are made based on limited data. This problem is compounded by the issue of patient variability mentioned earlier as it is easy to adhere to the treatment protocol with some patients whereas with others it is much more difficult. The second concern is that it is often the therapist who selects the sessions to be rated, a form of sampling that is highly likely to be biased. The third problem concerns the rater. Not infrequently ratings are made by someone who knows the therapist (such as the person providing supervision) and who might therefore already have a view about his or her ability, one which might colour their ratings.

#### The evaluation of standardised role plays

A third way of assessing therapists’ ability to implement treatments is through their performance in standardised role plays. This method is well established in medical education (where it is referred to as an “objective structured clinical examination” or OSCE; e.g., Newble, 2004), but it has only been employed to a limited extent to evaluate psychotherapeutic skills. This approach, however, has certain advantages over the therapy quality method of assessment, particularly as a means of evaluating the outcome of training. It will be discussed later.

## Recommendations regarding the assessment of therapy quality

There is only one way of directly assessing therapy quality which is to evaluate treatment sessions themselves. This requires rating sessions live or preferably assessing recordings of them.

As will be clear from the account above, substantial changes need to be made to the instruments currently available and their mode of use. The main changes are as follows:

i. Content – The features assessed need to encompass what are presumed to be the active components of the psychological treatment concerned and, depending upon the context, more generic psychotherapeutic elements. Each needs to be carefully defined, operationalised and specified in a manual for raters.
ii. Reliability – Rating schemes need to be devised that can be used reliably by raters representative of those who will utilise the instrument.
iii. Validity – This needs to be established both with respect to a total score, representing the overall quality of the session concerned, and a threshold score for judging the session to have been delivered sufficiently well for the treatment to be likely to achieve its expected effects.
iv. Protocol – This needs to specify the following:
  - The number of full sessions (or part-sessions) to be rated, and how they should be selected. If the goal is to evaluate the quality of a course of treatment it is important that a representative, and sufficiently large, sample of the treatment be assessed. With some treatments it might be important to specify that sessions be selected from specific sections of the treatment. Therapists should not be involved in selecting which sessions are rated (unless the assessment is for supervision purposes).
  - The choice of rater and the qualifications required to be one. The rater should be blind to the therapist’s identity (again, unless the assessment is for supervision purposes).

Progress has already been made in this regard: for example, D.M. Clark and colleagues have developed and validated an adaptation of the CTS (in German) to assess the quality of implementation of CBT for social phobia (personal communication). Further treatment-specific measures of therapy quality are needed. Such measures will be of great value in treatment research as they will provide much-needed means of assaying the standard of delivery of the treatment under investigation. They will also be of value in training therapists, both within and outside research settings. However, we suggest that such (therapy quality) measures are not suited to the assessment of therapist competence for reasons that will be discussed later.

## Part Two – Therapist Training How therapists learn to implement psychological treatments

There is a considerable demand for training in psychological treatments, but obtaining such training can be difficult. A two-stage approach is generally advocated for therapists with the relevant background experience (Beidas & Kendall, 2010; Sholomskas et al., 2005; Weissman et al., 2006). In the first stage the treatment is described and demonstrated (usually in the context of a “workshop” from an expert, typically lasting between 90 min and two days), and in the second the trainee uses the treatment under the supervision of a therapist who is proficient in the treatment concerned.

This method of training has not been well evaluated and it has several inherent shortcomings. Perhaps the most fundamental is that trainees have limited opportunity to see the treatment being practised – they can read about it; they can hear an expert talk about it; and they may see some brief video-recordings of segments of it; but few see the full treatment being implemented. Returning to the example of surgery, a parallel would be a surgeon being trained in a new surgical operation without ever seeing it performed. This would generally be thought to be a major concern. Like Sharpless and Barber (2009), we believe that it should be of no less concern that trainee therapists rarely see treatments practised from beginning to end. There are exceptions, of course – certain specialist training programmes provide opportunities for the extensive observation of treatment – but these are genuine exceptions.

An additional problem is that this method of training is not suited to the training of large numbers of therapists. Workshops from experts are costly and difficult to arrange, and few of those who attend will have the benefit of subsequent case supervision, either because there is no one available to provide it or because it is too expensive.

New methods of training are therefore needed, ones that are more cost-effective and scalable than the current method and, ideally, ones that are more effective. We suggest that internet-based training might be the answer.

## The use of the internet to enhance psychological treatment training

Over the past decade there has been mounting interest in delivering psychological treatment training electronically (e-Learning), either using CD-ROMs or, increasingly, using the internet (Cucciare, Weingardt, & Villafranca, 2008; Weingardt, 2004). The initial studies were relatively unsophisticated in their use of e-Learning technology, but recent research has begun to exploit more fully the potential of the internet. The emerging findings suggest that e-Learning can increase both knowledge about the treatment in question and, possibly, clinical skill. For example, two recent studies used interactive, media-enhanced, internet-based programmes to teach basic CBT skills to geographically dispersed trainees (Dimeff et al., 2009; Weingardt, Cucciare, Bellotti, & Lai, 2009). The programmes were completed in the trainees’ own time and comprised text, graphics, clinical vignettes and some recordings of acted therapist–patient scenarios. The results showed that the technology was readily used by the trainees and was highly acceptable to them. In both studies it resulted in knowledge acquisition, and in the study by Dimeff et al. (2009) this was greater than that obtained following a two-day training workshop although the methods were comparable with regard to the performance of a simple clinical skill.

This is just the beginning. Much greater use could be made of the training opportunities provided by the internet. Some of these are listed below:

i. Training material could be placed on dedicated clinically-rich websites. These could provide detailed but accessible information on the treatment’s indications and use, as well as its strategies and procedures. Thus they could duplicate and extend the information provided in face-to-face training workshops.
ii. The websites could include an extensive “library” of acted illustrations of the treatment, something that is impossible to provide in conventional workshops. The library could include complete treatments from beginning to end (involving different patients and therapists) accompanied by commentaries on them, as well as demonstrations of particular procedures.
iii. The websites could include formative tests of knowledge, including tests of clinical problem-solving, together with personalised guidance for further learning.
iv. The websites could incorporate inducements to complete the programme by providing ongoing positive feedback and by generating certificates (and scores) at various points.
v. The websites could prompt trainees to continue referring to them at intervals after the completion of the programme, perhaps by creating tailor-made refresher courses (with updated certification).

Training websites of this type would have a number of other advantages over conventional workshops:

i. They could be viewed (in whole or in part) on multiple occasions.
ii. They could be accessed wherever and whenever it suited the trainee and explored at a pace that the trainee could control.
iii. They could be evaluated by independent outside experts.
iv. They could be regularly and easily updated thereby reducing the time lag between the development of treatment advances and their dissemination to therapists.

There are several ways in which such websites could be used to provide training, two of which are described below.

### Internet-enhanced training

In this type of training access to a website of the type described would replace the initial training workshop and would alter the form of subsequent case supervision.

The training would start with trainees having several weeks to work their way through the core material on the website including both the factual material and the core clinical demonstrations. Once this had been done and the trainee had attained a satisfactory score on embedded questionnaires, the trainee would move on to receive a modified form of case supervision in which the supervisor would make extensive and repeated reference to the website and, in particular, to the library of acted clinical illustrations. The supervisor would not need to be an expert in the treatment: rather, he or she would need to be good at providing “*web-centred supervision*”. This would require that the supervisor have good generic supervisory skills, be familiar with the disorder being treated and the general class of psychotherapy in question, and have intimate knowledge of the website. Thus the supervisor’s role would differ from that in conventional supervision. Instead of providing expert guidance themselves, supervisors would help trainees solve problems and enhance their practice by referring to the information, guidance and illustrations on the website. Thus their role would be somewhat equivalent to that of a “facilitator” (as against that of a therapist) in guided self-help (Carter & Fairburn, 1998; Fairburn & Carter, 1997).

We predict that this form of training would be as effective as conventional training, if not more so, because of the availability of the clinically-rich website and the repeated use of it in supervision. It would certainly be less expensive and more scalable as there would be no need for an initial workshop and the supervisor would not need to be a specialist in the treatment concerned.

### Internet-alone training

An alternative to internet-enhanced training would be internet-alone training. This would involve trainees obtaining their entire training from the website. There would be no initial workshop and no subsequent case supervision. Internet-alone training would therefore be far less costly than conventional training. It would also be immensely scalable. On the other hand it would be likely to be less effective given the absence of case supervision. Nevertheless, if a worthwhile subgroup of trainees could train themselves this way, internet-alone training would be of enormous value as a means disseminating treatment expertise.

The characteristics of these two forms of training are summarised in Table 1 where they are compared with those of conventional training.

## Towards evidence-based training

There has been limited research on therapist training yet the training of therapists is as amenable to research as are psychological treatments themselves, the design of choice being the randomised controlled trial (RCT). Unfortunately, the RCTs undertaken to date have had important shortcomings (Herschell et al., 2010; Rakovshik & McManus, 2010). These include the following:

#### Treatments studied

The RCTs have focused on relatively simple interventions (behavioural treatments for phobias; various interventions for substance abuse; group skills training in dialectical behaviour therapy) whereas many of the leading evidence-based psychological treatments (such as those for depression, eating disorders and anxiety disorders) are more complex and may therefore be harder to learn.

#### Trainees

The trainees have mostly comprised substance abuse counsellors, trainee nurses and medical students. It is not clear that findings obtained with such trainees will generalise to other types of therapist or to those with more experience delivering psychological treatments.

#### Outcome measures

As noted earlier, little attention has been paid to the assessment of the outcome of training (therapist competence). There has been reliance on non-standardised measures of knowledge and relatively crude assessments of psychotherapeutic skill.

#### Statistical power

The studies have mostly been underpowered.

#### Maintenance of training effects and value of subsequent supervision

Few studies have assessed the maintenance of clinical skills following training. Just two of the RCTs have examined the value of clinical supervision (Mannix et al., 2006; Miller, Yahne, Moyers, Martinez, & Pirritano, 2004).

In light of these shortcomings, few, if any, firm conclusions can be drawn from the findings to date.

Most of these methodological problems could be easily addressed. Training research could focus on more sophisticated treatments than those studied to date, and therapists with a variety of backgrounds could serve as the trainees: indeed, this would be of great value as the trainee characteristics predictive of a successful outcome are not known (Rakovshik & McManus, 2010). Future RCTs could also have sufficiently large sample sizes to be adequately powered and they could incorporate follow-up periods (with and without case supervision) to allow the maintenance of training effects to be determined. A major challenge, however, would be the evaluation of the outcome of training; that is, therapist competence. We conclude this article by returning to this topic.

## Recommendations regarding the assessment of therapist competence

As defined above, therapist competence refers to the “the extent to which a therapist has the knowledge and skill required to deliver a treatment to the standard needed for it to achieve its expected effects.” Thus measures of therapist competence need to be capable of assessing the requisite knowledge and the therapist’s ability to apply this knowledge in clinical practice. In addition, if such measures are to be used to evaluate the effects of training they need to be suitable for repeated use (e.g., before training, after training and at periods thereafter).

In principle, the assessment of knowledge is relatively straightforward. Multiple-choice questions of various formats could be used to assess both the relevant factual information and clinical problem-solving. The evaluation of psychotherapeutic skill is more complex. To date the main method used has involved rating treatment sessions but, for largely practical reasons, this “therapy quality” method is not suited to the testing of the skill component of therapist competence – and this would apply even if improved session-rating measures were available. This is because a wide range of sessions would need to be evaluated in order to sample the treatment’s main strategies and procedures, and sessions would need to be selected from patients of varying difficulty. This is rarely feasible post-training and almost impossible pre-training.

Instead, we suggest that a role play-based method of assessment would be preferable. It would involve the trainee being the “therapist” with a simulated patient who would enact a series of prepared clinical scenarios. These would be selected from a battery of such scenarios and, in marked contrast with the therapy quality method of assessment, they could be chosen to be representative of the treatment’s core strategies and procedures, and to involve patients of varying difficulty.

As yet, this method has not been used, the closest approximation being the assessment protocol developed by Sholomskas and Carroll (2006). It involved trainees in twelve-step facilitation being asked to interact with a simulated patient and demonstrate five treatment skills over about 1 h. Recordings of the role-plays, made both before training and three weeks later, were subsequently assessed by raters who were blind to the participant’s training condition.

The major difficulty in developing such a method of assessment would lie in creating the scenarios and in devising a reliable way of rating the trainee’s performance. The validation of the resulting score or scores (and the threshold for judging the trainee to be “competent”) would need to be against the content of the relevant treatment manuals, the views of experts in the treatment concerned, and the performance of therapists of known levels of ability. The relationship of the score to patient outcome would also need to be determined. Although difficult to accomplish, work of this type needs to be undertaken if evidence-based treatments are to be effectively and efficiently disseminated.

## Figures and Tables

**Table 1:** Characteristics of conventional training, internet-enhanced training and internet-alone training.

	Conventional training	Internet-enhanced training	Internet-alone training
*Format*			
Initial workshop	Yes	No	No
Access to training website	No	Yes	Yes
Case supervision	Yes	Yes	No

*Strengths and weaknesses (compared with conventional training)*			
Likely effectiveness	–	As effective or more effective	Less effective
Likely cost^a^	–	Less costly (as no workshop and a less specialist supervisor)	Much less costly (as no workshop and no case supervision)
Scalability	–	More scalable (as no workshop and a less specialist supervisor)	Markedly more scalable (as no workshop and no case supervision)

a This does not take account of the initial cost incurred creating the website.

## Footnotes

1 We use the term “psychotherapist” to refer to professionals who specialise in providing psychological treatments, whatever their professional background. Along similar lines, we use the terms “psychological treatment” and “psychotherapy” interchangeably, as fits the context.

2 We prefer the term “therapy quality” over “treatment fidelity” and “treatment integrity” as, in our opinion, it conveys more simply and clearly what is meant.
